# SARS‐CoV‐2: March toward adaptation

**DOI:** 10.1002/jmv.26233

**Published:** 2020-07-11

**Authors:** Francesca Benedetti, Maria Pachetti, Bruna Marini, Rudy Ippodrino, Massimo Ciccozzi, Davide Zella

**Affiliations:** ^1^ Department of Biochemistry and Molecular Biology, School of Medicine, Institute of Human Virology University of Maryland Baltimore Maryland; ^2^ Department of Physics, Elettra Sincrotrone Trieste University of Trieste Trieste Italy; ^3^ Ulisse BioMed Trieste Italy; ^4^ Medical Statistic and Molecular Epidemiology Unit University of Biomedical Campus Rome Italy; ^5^ Global Virus Network Baltimore Maryland

## 1 INTRODUCTION

The worldwide spread of severe acute respiratory syndrome coronavirus 2 (SARS‐CoV‐2), the novel human pathogen, first detected in China quickly became a global health emergency, culminating with the World Health Organization publicly proclaiming the SARS‐CoV‐2 outbreak as a pandemic (11 March 2020). So far, more than 7 million people have been infected and more than 400 thousand have died, causing profound economic disruption and unsettling consolidated human traditions. SARS‐CoV‐2 is an enveloped +single‐stranded RNA virus, belonging to the *Betacoronavirus* genus, which includes two other RNA viruses that have caused recent important epidemics: SARS caused by SARS‐CoV and the Middle East respiratory syndrome (MERS) caused by MERS‐CoV. SARS‐CoV and MERS‐CoV have caused more than 10 000 cumulative cases in the past 2 decades, with mortality rates of 9.6% for SARS‐CoV and 37% for MERS‐CoV, respectively.^1^, ^2^, ^3^, ^4^

Viral fitness of RNA viruses is influenced by their very high frequency of mutation, which, in turn, can affect infection speed and evolution rate.^5^ However, the resulting genetic variability needs to be balanced between the maintenance of genetic information integrity and survival within the host.^6^, ^7^, ^8^ The viral genome mutagenic process is depended on several factors, including viral enzymes responsible for nucleic acids replication, which may have few or no proofreading and/or postreplicative nucleic acid repair capability. In addition, host enzymes, spontaneous nucleic acid damages due to physical and chemical mutagens, and recombination events can contribute to the genomic variability, together with particular genetic elements responsible for the production of new variants. Mutation rates are modulated also by additional factors such as determinants of the template sequence and structure involved in viral replication.^5^ In most viruses, RNA polymerase lacks proofreading capability, with some exceptions such as *Nidovirales* order (to which the *Betacororonavirus* belongs). In *Nidoviruses*, complex machinery originated as cleavage products of the viral ORF1a and ORF1b, composed of a polymerase (RNA‐depended RNA polymerase) and nonstructural proteins, is responsible for virus replication and transcription.^9^, ^10^ Biological characterization of viral mutations provide precious insights for assessing viral drug resistance, immune escape, and pathogenesis‐related mechanisms. In addition, viral mutation studies are crucial for designing new vaccines, antiviral drugs, and diagnostic assays.

Several reports result show that SARS‐CoV‐2 is rapidly moving across countries, and new mutation hotspots are emerging in different parts of the genome.^11^, ^12^, ^13^, ^14^, ^15^, ^16^, ^17^, ^18^ Such variability indeed has implications for control of the pandemic and potential emergence of viral strains with different characteristics, including increased or reduced infectivity and lethality. Although SARS‐CoV‐2 is less lethal than MERS‐CoV, up to 20% of the infected people develop rapidly a severe disease characterized by interstitial pneumonia and acute respiratory distress syndrome that can ultimately lead to death. This is particularly evident in elderly and in people with underlying medical conditions.^19^ However, most of the patients remain asymptomatic or develop mild symptoms, like fever and dry cough, followed then by breathing difficulties (dyspnea), and bilateral ground‐glass opacities on chest computed tomography scans, indicating that the target cells are located in the lower airways.^20^

Determination of infected subjects and mortality calculations during the epidemics are difficult. During the initial period of the epidemic, many patients were diagnosed with coronavirus disease 2019 (COVID‐19) only after developing critical illness or even at the time of death, whereas asymptomatic or paucisymptomatic patients were untested, leading to an underestimation of the denominator.^21^ Additional significant biases affect mortality curves: to name a few, the parameters used for death counting, the rigidity of lockdown measures, and population age. Over time, countries started adopting better policies for diagnostic polymerase chain reaction testing and lockdown strategies, and, consequently, the spread of the virus was better monitored and the data were more carefully determined.

Recent evidence indicate that the social distance measures are progressively having a positive impact on reduction of mortality,^22^ and these measures can also be helped by the seasonal increase in temperatures and humidity,^23^, ^24^ like already observed for other respiratory viruses.^25^, ^26^, ^27^ In addition, several mutated strains of SARS‐CoV‐2 are constantly emerging,^28^, ^29^ while the infectivity rates and COVID‐19‐associated number of deaths are decreasing.^30^

The extent of such mutations and their effects on infectivity and mortality is being actively examined by population genetic analyses of sequenced genomes of SARS‐CoV‐2. While some studies suggested that specific mutations may be correlated with increased transmissibility,^17^ other studies showed that deletions in SARS‐CoV‐2 genomes are emerging^28^, ^29^ that may potentially reduce virus replication, similarly to what observed in the case of SARS‐CoV.^31^, ^32^, ^33^ These latter observations are not surprising and may be more appropriately represent the current stage of the pandemic. In fact, the final objective of viruses such as SARS‐CoV‐2 is to reach a point of equilibrium with their host and become endemic, thus allowing spreading in the absence of a high mortality rate.

## 2 CONCLUSION

Though possible, it is unlikely that the early viral mutations observed in Europe and then in the US after the virus emerged from China radically influenced its fitness. Moreover, their contribution to lethality is difficult to determine, since at the beginning of a pandemic event, the virus is likely to be very aggressive and its sequences would tend to be more homogenous. On the other hand, the early mutations we observed in the viral polymerase gene could have affected its processivity and fidelity, further increasing its mutation rate and the generation of viral clades progressively more heterogeneous.

The emergence of subsequent specific patterns of mutations, concomitant with the decline in case fatality rate, likely follows the principle of homoplasy and suggests a converged evolution due to the accumulation of mutations over time. This would, in turn, lead to the rapidly progressive and convergent adaptation of the virus to the human host. Additional confirmation and the biological significance of such mutations need to be determined. Nonetheless, it is tempting to speculate that they may contribute to the loss of virulence of SARS‐CoV‐2. These mutations may also be relevant for the design of new antiviral agents and vaccines.

From a public health perspective, these data are important in the case SARS‐CoV‐2 reemerges in late fall or early winter of 2020, or another respiratory virus appears in the distant future. Timely viral detection, more precise, and reliable tracking methods leading to swift isolation of exposed and infected subjects together with the prompt implementation of measures of social distancing may be complemented with weather pattern analysis and viral sequence analysis, to help to shape more focused interventions for the quick recognition and early containment of new emerging clusters of infection.

## CONFLICT OF INTERESTS

The authors declare that there are no conflict of interests.
